# Modeling the cell biology of monogenetic intestinal epithelial disorders

**DOI:** 10.1083/jcb.202310118

**Published:** 2024-04-29

**Authors:** Izumi Kaji, Jay R. Thiagarajah, James R. Goldenring

**Affiliations:** 1 Section of Surgical Sciences, Vanderbilt University Medical Center, Nashville, TN, USA; 2 Department of Cell and Developmental Biology, Vanderbilt University School of Medicine, Nashville, TN, USA; 3 Epithelial Biology Center, Vanderbilt University School of Medicine, Nashville, TN, USA; 4 Division of Gastroenterology, Hepatology and Nutrition, Boston Children’s Hospital, Harvard Medical School, Boston, MA, USA; 5 Congenital Enteropathy Program, Boston Children’s Hospital, Boston, MA, USA; 6 Harvard Digestive Disease Center, Boston, MA, USA; 7 Nashville VA Medical Center, Nashville, TN, USA

## Abstract

Monogenetic variants are responsible for a range of congenital human diseases. Variants in genes that are important for intestinal epithelial function cause a group of disorders characterized by severe diarrhea and loss of nutrient absorption called congenital diarrheas and enteropathies (CODEs). CODE-causing genes include nutrient transporters, enzymes, structural proteins, and vesicular trafficking proteins in intestinal epithelial cells. Several severe CODE disorders result from the loss-of-function in key regulators of polarized endocytic trafficking such as the motor protein, Myosin VB (MYO5B), as well as STX3, STXBP2, and UNC45A. Investigations of the cell biology and pathophysiology following loss-of-function in these genes have led to an increased understanding of both homeostatic and pathological vesicular trafficking in intestinal epithelial cells. Modeling different CODEs through investigation of changes in patient tissues, coupled with the development of animal models and patient-derived enteroids, has provided critical insights into the enterocyte differentiation and function. Linking basic knowledge of cell biology with the phenotype of specific patient variants is a key step in developing effective treatments for rare monogenetic diseases. This knowledge can also be applied more broadly to our understanding of common epithelial disorders.

## Teaser

Goldenring et al. review recent findings in cell, organoid, and animal models for monogenic diseases causing congenital diarrhea and enteropathies (CODEs).

## Introduction

Human diseases that arise from heritable changes in single genes are rare, but often result in severe symptoms and phenotypes. These monogenetic disorders commonly manifest early in life and show a wide range of pathology that can be cell-type or organ-specific or have broader multi-system implications. At a cellular level, monogenetic disorders can lead to profound alterations in cell structure and function. Monogenetic intestinal epithelial disorders, also known as congenital diarrheas and enteropathies (CODEs), are a group of rare diseases that result from mutations in genes that primarily affect intestinal epithelial cell function. Patients with CODE disorders generally present with infantile-onset diarrhea and poor growth and often require intensive fluid and nutritional management. Genes involved in CODE disorders relate to broad areas of intestinal epithelial function, structure, and development.

### Identification of monogenetic intestinal epithelial disorders

Most CODE disorders are characterized by loss-of-function gene variants and are inherited in an autosomal recessive manner. There are only a few disorders that result from dominant active or dominant negative variants (Babcock et al., 2023; Janecke et al., 2016). Although manifestations of CODE gene variants are often present in the neonatal period, until recently, many infants did not receive a definitive genetic diagnosis due to a lack of availability and awareness around appropriate genetic testing. The advent of low-cost whole exome and genome sequencing (WES/WGS) and gradually increased recognition of these disorders by pediatric medical practitioners has led to considerable advances in diagnosis and the identification of the causative gene variants (Elkadri, 2020; Mantoo et al., 2021; Russo, 2020; Thiagarajah et al., 2018; Younis et al., 2020). With rapid clinical exome sequencing now available in many pediatric medical centers, the numbers of causative genes and types of gene variants have quickly expanded. This expansion has led to an increasing need for functional assessments of disorder-associated genes to shed light on the cellular pathobiology as well as to provide potential avenues for disease therapy. Using a variety of cell and animal models, we now have the capability to interrogate disease pathophysiology using a personalized cell biology approach that can reveal and recapitulate physiological deficits and allow testing of potential treatment strategies. In the subsequent discussion, we use examples of recent advances in our understanding of specific CODE disorders to illustrate how these approaches can provide insights into intestinal epithelial cell biology.

### Intestinal epithelial structure and function

**Figure 1.**
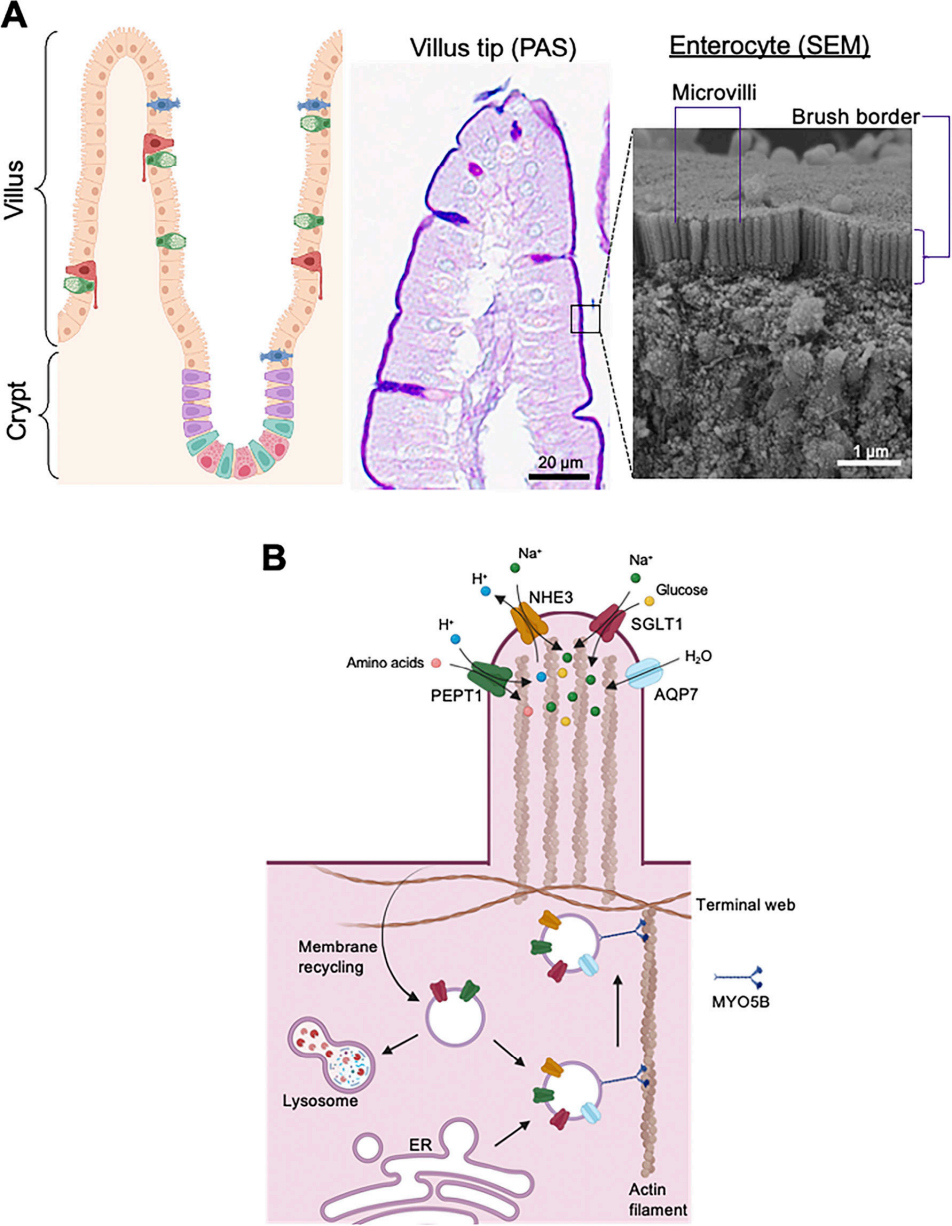
**Intestinal brush border structure and function. (A)** Intestinal epithelium consists of a variety of cell types lining the luminal surface along the crypt-villus axis of mucosal architecture. PAS staining of tissues visualizes glycoproteins as dark purple and represents a good histological marker for mature brush border and goblet cells. Individual microvillus structure is demonstrated by scanning electron microscopy (SEM). **(B)** Absorptive transporters for nutrients and water in the microvillus. After protein synthesis, the apical membrane transporters are trafficked by post-Golgi vesicles and inserted into the membrane. Apical membranes containing some transporters can be endocytosed and either recycled or fused with lysosomes to facilitate degradation. This figure was created with Biorender.

The intestinal epithelium is organized as a single layer of cells that line the luminal (outside) surface of the intestinal tract. In the small intestine, this layer is architecturally characterized by glands (crypts) and finger-like projections (villi) (Fig. 1 A). Intestinal epithelial cells are generated from pluripotent stem cells that reside in the base of crypts and are marked by the receptor protein Lgr5 (Barker et al., 2007; Gehart and Clevers, 2019). These stem cells give rise to progenitor cell populations that ultimately differentiate into a wide range of different epithelial subtypes including secretory cells, such as goblet and enteroendocrine cells, as well as columnar absorptive cells referred as enterocytes (Santos et al., 2018). The surface epithelium in the small intestine plays several primary roles, including fluid and nutrient transport and maintenance of barrier function. Intestinal fluid absorption and secretion occur secondary to electrolyte flux across the epithelium facilitated by an array of transport and channel proteins. Fluid absorption is primarily driven by transepithelial sodium transport and fluid secretion by chloride secretion (Larsen et al., 2002; Rao, 2019). Diarrhea is caused by loss of normal fluid absorption and/or excessive fluid secretion usually secondary to altered function in electrolyte and nutrient transporters across the intestinal epithelium. The lumen-facing or apical membrane of enterocytes is elaborated by a well-organized network of F-actin-based microvilli that markedly expand the surface absorptive area (Mooseker and Tilney, 1975) (Fig. 1 A). Microvilli harbor major sodium transporters NHE3 and SGLT1 that are responsible for the majority of sodium absorptive function (Hoogerwerf et al., 1996; Hwang et al., 1991). Microvilli also harbor chloride channels including CFTR, which is the major pathway for chloride secretion (Tousson et al., 1996). The microvilli additionally contain more specialized nutrient transporters as well as digestive enzymes (e.g., dipeptidyl peptidase IV, sucrase isomaltase, and alkaline phosphatase) (Ono, 1975; Yoshioka et al., 1991). These functionally mature microvilli are also referred to as the brush border, since its structure appears like the bristles of a paintbrush (Fig. 1 A). Maintenance of transport proteins on microvilli is accomplished through specialized polarized endocytic trafficking pathways present in columnar epithelial cells.

### CODE genes and epithelial function

Variants involved in CODE disorders encompass several broad categories of epithelial function, including transporters, brush border enzymes, structural proteins, as well as proteins involved in intracellular trafficking. Patients with CODEs commonly show blunted intestinal villi and immature brush borders in duodenal biopsies, and a variety of defects in nutrient absorption and metabolism (Babcock et al., 2023; Thiagarajah et al., 2018). Many CODE patients require life-long parenteral nutrition and/or multiorgan transplantation, interventions associated with multiple complications and morbidity. A major impetus therefore for understanding the molecular basis of CODEs is the development of disease-specific treatment options.

Fluid absorption in the intestine is critically dependent on the function of apical transporters, such as sodium-dependent glucose cotransporter 1 (SGLT1/SLC5A1), sodium-proton exchanger 3 (NHE3/SLC9A3), down-regulated in adenocarcinoma (DRA/SLC26A3), solute carrier organic anion transporter 2A1 (SLCO2A1), and the apical sodium/bile acid transporter (ASBT/SLC10A2). Consequently, several CODE disorders result from biallelic gene variants that lead to the loss or disabling of these transporters, with subsequent induction of varying levels of diarrhea. SGLT1 serves as a critical driving force for water absorption in the small intestine, and its mutations cause glucose-galactose malabsorption (GGM), leading to substrate-induced diarrhea from dietary glucose and galactose, but not fructose (Martín et al., 1996; Meinild et al., 1998). The apical ion exchangers, NHE3 and DRA, are important for sodium and chloride absorption, respectively. NHE3 deficiency and mutations have been identified in congenital sodium diarrheal patients (Booth et al., 1985; Janecke et al., 2015), as well as DRA mutations in congenital chloride diarrhea (Höglund et al., 1996). ASBT expression is limited to the distal portion of the small intestine (ileum), and bile acid diarrhea is linked to ASBT mutations and ileal resection (Qie et al., 2021). In addition to these ion channels, diarrhea and malabsorption, albeit generally less severe, may also emanate from deficits in mature brush border enzymes such as sucrase isomaltase (SI) (Geng et al., 2014).

While mutations in apical nutrient transporters have clear implications for malabsorption, other CODE disorders may have less direct implications. Disabling mutations in acyl-CoA:Diacylglycerol *O*-acyltransferase 1 (DGAT1), an enzyme that catalyzes triglyceride synthesis from diacylglycerol and free fatty acids, cause infantile diarrhea. By exome sequencing in patients, several biallelic mutations in DGAT1 have been identified that lead to its unfolding and degradation (Garcia-Castillo et al., 2017; Gluchowski et al., 2017; van Rijn et al., 2018; Ye et al., 2019). DGAT1 is essential for dietary fat absorption and processing in intestinal enterocytes, and another isoform DGAT2 expression is primarily limited to liver and adipose tissues in humans (van Rijn et al., 2018). CODE patients with DGAT1 deficiency demonstrate prominent diarrhea and protein loss beginning in their first weeks of life, requiring cessation of oral feeding, and use of total parenteral nutrition (Haas et al., 2012; Schlegel et al., 2018). Interestingly, a recent study indicates that Rotavirus-induced diarrhea is induced by DGAT1 degradation in the infected enterocytes (Liu et al., 2023). In addition to fat absorption, DGAT1-mediated lipid processing in enterocytes is likely critical for overall brush border function. Initial biopsies of patients with DGAT1 mutations show partial villus blunting and disrupted brush border structures (Schlegel et al., 2018; van Rijn et al., 2018). Similar to other CODE patients, DGAT1 deficiency patient tissues demonstrate the mislocalization of apical nutrient transporters that is a direct cause of malabsorption and diarrhea. Nevertheless, molecular mechanisms for how DGAT1 deficiency alters brush border protein trafficking have not been fully elucidated.

### Intracellular trafficking: A major pathway involved in CODE

Several severe CODE disorders involve mutations that impact key regulators of intracellular vesicle trafficking. The most well-known of these disorders is Microvillus Inclusion Disease (MVID), which results from inactivating mutations in the vesicular motor protein, myosin VB (MYO5B) (Erickson et al., 2008; Müller et al., 2008). MYO5B interacts through its cargo-binding tail domain with a number of Rab small GTPases, including Rab11a, Rab11b, Rab25, and Rab8a, and regulates transcytosis and apical membrane recycling in epithelial cells (Lapierre et al., 2001; Roland et al., 2007). Rab proteins are thought to act as molecular zip codes determining distinct pathways for vesicle trafficking through their interactions with specific mediators (Novick and Zerial, 1997). MVID patients experience life-threatening diarrhea usually beginning in the first week of life. Histological and ultrastructural analyses demonstrate severe villus blunting, loss of apical microvilli, and the variable presence of intracellular microvillus inclusions, which are pathognomonic for the disease (Cutz et al., 1989; Davidson et al., 1978).

In the majority of CODE disorders, loss of epithelial function in patient tissues coincides with alterations in transporters and enzymes in the microvilli, as well as changes in the distribution of cytoskeletal elements and vesicle-trafficking proteins. In MVID, several studies have demonstrated the loss of sodium transporters and enzymes from the apical membranes, suggesting that the major cause of diarrhea is an inability to absorb sodium and water (Ameen and Salas, 2000; Knowles et al., 2014). MYO5B has been studied extensively as a regulator of apical recycling and transcytosis and found to regulate the localization of several apical transporters (Engevik and Goldenring, 2018). The absence of active MYO5B leads to transporters and enzymes failing to reach to the apical membrane and consequent abnormal localization in the subapical region of the cytoplasm or degradation in lysosomes (Fig. 1 B). One general question in epithelial cell biology has focused on how many pathways may be available for trafficking to the apical membrane and whether these trafficking mechanisms are all tied to post-Golgi vesicles or use fusion with endosomal recycling system components (Fig. 1 B). Immunohistochemical assessment of patient biopsies with loss of MYO5B function has now demonstrated that loss of apical protein trafficking is not global, since the apical localization of the chloride channel, CFTR, is preserved in patient enterocytes (Engevik et al., 2018; Kravtsov et al., 2016). It therefore appears likely that individual cargoes targeted to the apical brush border are processed through both direct transport from post-Golgi pathways and alternative pathways that likely include intermediate compartments of the apical recycling system.

Mutations in different proteins often manifest similar pathology in patients when the proteins are connected through common membrane trafficking pathways. In the case of apical protein trafficking, defects in the unc-45 myosin chaperone A (UNC45A), syntaxin 3 (STX3), and syntaxin-binding protein2 (STXBP2/MUNC18-2) also result in cellular phenotypes similar to MYO5B loss-of-function (Duclaux-Loras et al., 2022; Wiegerinck et al., 2014). Several case reports suggest that loss-of-function mutations in these trafficking-associated proteins cause a variant of the phenotype found in classical MVID due to MYO5B (Alsaleem et al., 2017; Duclaux-Loras et al., 2022; Hackmann et al., 2013; Stepensky et al., 2013; Vogel et al., 2017; Wiegerinck et al., 2014). Patients with mutations in these proteins can exhibit microvillus inclusions or lateral microvilli in their duodenal biopsies, indicating apical trafficking issues similar to those induced by MYO5B mutations.

UNC45A is a ubiquitously expressed co-chaperone protein and essential for the proper folding of non-muscle type II myosins and MYO5B. Functional loss of UNC45A causes multiorgan failure, osteo-oto-hepato-enteric (O2HE) syndrome (Esteve et al., 2018; Li et al., 2022). Several biallelic variants in UNC45A have been identified in O2HE patients who suffered from MVID-like congenital diarrhea. These patients with UNC45A deficiency consistently demonstrate the altered microvillus morphology and mislocalized brush border proteins, such as NHE3 and DRA, leading to osmotic diarrhea (Duclaux-Loras et al., 2022; Esteve et al., 2018). STX3 is a soluble N-ethylmaleimide-sensitive factor attachment protein receptor (SNARE) protein, and STX3 loss causes metabolic acidosis and chronic diarrhea (Alsaleem et al., 2017; Wiegerinck et al., 2014). The patients’ tissues and biopsy-derived organoids with STX3 variants demonstrate mislocalized microvilli and abnormal PAS+ vesicles in blunted villi, albeit their brush border actin structure is closer to normal than that in tissues with MYO5B defects. STXBP2 is a regulatory protein for the SNARE complex (Liu et al., 2007). Mutations in STXBP2 cause familial hemophagocytic lymphohistiocytosis type 5 (FHL5), an immune disorder also associated with chronic diarrhea (Stepensky et al., 2013; Vogel et al., 2017). Analysis of small intestine tissue histology in FHL5 patients reveals short microvilli lacking the brush border proteins, NHE3 and CD10. Together, these observations in patients suggest that UNC45A and SNARE complexes are important for establishing a functional brush border and absorption of water and nutrients from the lumen of the intestine. Identification of human pathological mutations in these vesicular trafficking-related proteins has therefore provided a window into a critical pathway of interconnected regulators of polarized trafficking in intestinal epithelia.

It is important to note that patient tissue samples from these CODE conditions provide a critical source for comparison with in vitro and mouse modeling efforts. While these modeling efforts seek to replicate the phenotypes of patients, one must also consider that patients may be subjected to iatrogenic influences that may impact tissue presentation. For example, all of these patients are placed on total parenteral nutrition and often receive no oral feeding to control diarrhea. A lack of oral feeding itself usually results in villus atrophy (Goldstein et al., 1985), so patient tissue presentations may reflect a combination of primary disease implications along with secondary treatment outcomes. These considerations need to be factored into efforts to validate patient phenotypes with cell or animal-based modeling of disease.

## Modeling CODEs: A window into epithelial function

**Figure 2.**
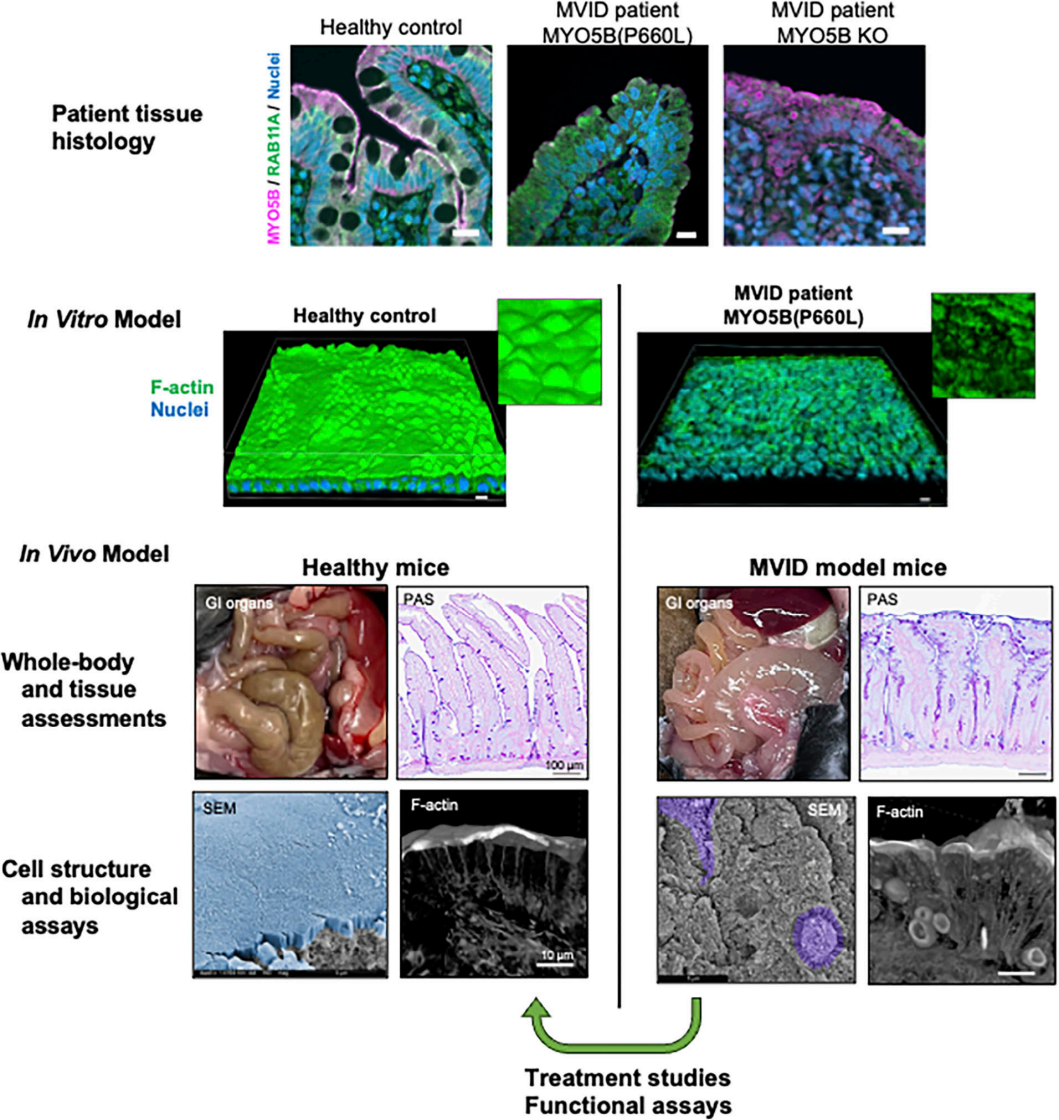
**A pathway for investigation of the cellular physiology of CODE disorders exemplified by investigations of microvillus inclusion disease caused by inactivating mutations in MYO5B.** Phenotypic patterns for aberrant apical trafficking are identified in patient tissue (duodenum). These patterns can be recapitulated in human patient-derived enteroids grown on Transwell filters in ALI conditions to induce differentiation (in vitro model). In ALI cultures, normal enterocytes demonstrate a dense actin-rich brush border, while MVID patient enteroid monolayers show disrupted microvillar structures. In vivo models of MYO5B deletion or loss-of-function also recapitulate the histology of MVID with the formation of microvillus inclusions and aberrant brush border structure (in SEM images, blue pseudo-coloring shows normal microvilli at left, while purple pseudocolor shows the presence of microvillus inclusion in MYO5B(G519R) mutant intestine). Reversal of aberrant phenotypes can then be used in vitro and in vivo to develop putative therapies.

Patients harbor a wide variety of different variants in specific CODE disorder genes such as MYO5B and other genes involved in polarized intracellular trafficking. The resulting effects on the corresponding proteins range from complete loss to subtle alterations in function, with similarly widely varying effects on cellular phenotype. Therefore, investigating the functional implications of both total loss of protein as well as protein domain-specific alterations can potentially provide a wealth of information on protein structure–function relationships. These investigations have utilized a range of methods including assessment of patient tissue samples, and modeling specific patient variants in transformed cell lines, patient-derived organoids, and mouse models. As shown in Fig. 2, MVID caused by loss-of-function MYO5B variants has been studied in multiple models and experimental systems. Characterization of these variants has focused on ultrastructural alterations as well as deficits in transporter trafficking as documented in immunostaining as well as functional assay of transporter function. This section discusses the utility and limitation of different disease model approaches, focusing on modeling of loss of MYO5B function as an example.

### Modeling CODE diseases in vitro with immortal cultured cell lines

A common approach for modeling mutations of putative disease-causing proteins is the use of immortal cell lines derived from cancers. Studies have often utilized the ability to perform shRNA knockdown or CRISPR deletion to develop cell lines with either reduced expression or loss-of-function. In the case of MVID, most studies have used CaCo2-BBe cells, a human colon cancer cell line that has properties more consistent with small intestinal epithelial cells than colonocytes. This cell line has the ability to assemble polarized apical microvilli, especially when cultured on permeable filters (Tyska et al., 2005; Tyska and Mooseker, 2004). Several investigators have documented changes in microvillus formation and organization following knockdown of MYO5B expression in CaCo-2BBe cells (Knowles et al., 2014; Kravtsov et al., 2014; Thoeni et al., 2014). In this model, reexpression of GFP-tagged wild-type MYO5B could rescue microvilli structure, while reexpression of MYO5B(P660L), the mutant present in Navajo MVID patients, resulted in the formation of microvillus inclusions (Knowles et al., 2014). The missense P660L mutation is the only MYO5B mutation that has been studied mechanistically. P660L confers a rigor status to MYO5B motor function, implicating binding to actin filaments, and resulting in failure to complete a full motor stroke. These studies also evaluated rescue with MYO5B sequences harboring mutations that prevent the binding of either Rab11a or Rab8a (Knowles et al., 2014), which were identified in previous work in cultured epithelial cell lines (Lapierre et al., 2001; Roland et al., 2007, 2011). Loss of Rab11a binding to MYO5B was linked with a formation of microvillus inclusions, while loss of Rab8a binding to MYO5B was associated with defects in brush border trafficking (Knowles et al., 2014). Consistent with this, RAB8A knockdown CaCo-2 cells results in reduced sodium-dependent vitamin transporter function due to impaired apical trafficking (Subramanian et al., 2013). Knockdown of RAB11A in CaCo2-BBe cells disrupts cell polarity and induces mislocalized microvilli in basolateral domain (Knowles et al., 2015). Despite these related cellular phenotypes in cell lines, no mutations in either RAB11a or RAB8A proteins have been identified in patients presenting with MVID-like disease. It is likely that loss-of-function mutations in these RAB proteins lead to early lethality.

While studies in Caco2-BBe cells were able to delve into MYO5B-dependent effects on microvilli formation, insights into transporter trafficking were limited as these cells do not express and localize many of the key apical transporters, including NHE3 and SGLT1, at levels seen in native tissue. While some subclones of CaCo2 cells have been isolated with demonstrable endogenous NHE3 expression (Janecki et al., 1999), the majority of studies assessing intestinal transport have been performed using stable-transfected clones over-expressing specific transporters (Zhao et al., 2004). The lack of physiologically appropriate transporter expression in cancer cell line models has therefore limited cell biological interpretation of homeostatic versus disease-related changes in their trafficking. Nevertheless, these systems can provide insights into the aspects of trafficking that are better replicated such as some brush border enzymes. Furthermore, cell lines may provide an alternative for modeling when mouse models are not relevant to the human biology. Such is the case for DGAT1, where the homologous DGAT1 gene and its cousin DGAT2 are not expressed in rodents as in humans, and deletion of one or other of DGAT genes in mice does not phenocopy the syndrome in humans (Chen and Farese, 2005; Gluchowski et al., 2017; Vujić et al., 2020). Nevertheless, DGAT1 knockdown could be performed in CaCo2-BBe cells with recapitulation of the impairment in brush border integrity and amelioration of the phenotype with culturing without lipids in the apical media (Schlegel et al., 2018).

### Development of in vivo animal models for CODE disorders

Total and tissue-specific knockout mice are useful to study the cell biology and pathogenesis of loss-of-function mutations in monogenic diseases in vivo. Knockout mouse strains have been developed by (1) modifying embryonic stem (ES) cells using artificial DNA vectors to disrupt an exon and (2) through a random (non-targeted) genome disruption. Both methods can lead to whole-body knockouts and the ES cell modifications can also lead to the development of conditional knockout alleles by an establishment of floxed exon flanking sequences. In the case of the gene-trap method, homozygous gene disruption in MYO5B leads to early lethality within the first day of life (Cartón-García et al., 2015). Germline MYO5B knockout derived from ES cells demonstrated a similar early lethality on the C57BL/6 background, but breeding these mice onto an outbred CD1 background increased viability up to 5 days postnatal (Weis et al., 2016). In both these germline knockout mouse models, deficits in apical membrane microvilli, numerous microvillus inclusions, and losses in apical transporters were identified in the small intestine. Notably, heterozygote mice showed a healthy phenotype, similar to the findings in humans.

Several groups have produced both constitutive and inducible intestine-targeted models for MYO5B loss through the creation of *Myo5b*-floxed alleles in mice (Schneeberger et al., 2015; Weis et al., 2016). Constitutive deletion of MYO5B in the intestine with a Villin-Cre driver elicited a pattern of severe dehydration, loss of apical transporters, and microvillus inclusions within a few days after birth, similar to the phenotype in germline knockout mice (Weis et al., 2016). While the exact phenotypes in inducible intestinal deletion mice are not completely identical, induction of MYO5B deletion in adult mice with the Villlin-Cre^ERT2^ driver led to villus atrophy, microvillus inclusions, shortened microvilli, and loss of apical sodium transporters within 4 days of induction with tamoxifen (Engevik et al., 2018; Schneeberger et al., 2015; Weis et al., 2016). Thus, the mouse models could authentically replicate the clinical phenotype observed in MVID patients (Fig. 2). Moreover, the availability of the mouse models has allowed a more detailed characterization of the cell physiology of MYO5B loss. These studies have documented the expansion of lysosomes in enterocytes following MYO5B loss along with the accumulation of RAB11A-containing tubulovesicular elements (Engevik et al., 2019), all characteristics that have been observed in MVID patient tissues (Hess et al., 2021). Importantly, these mouse models have clarified a number of aspects of the cell biology of MVID. Examination of ion transport activity and transporter localization determined that the apical trafficking of the CFTR chloride channel was not affected by loss or patient-modeled variant of MYO5B (Burman et al., 2023; Engevik et al., 2018). Diarrhea observed in MVID patients therefore likely reflects both a lack of sodium absorption and continued chloride secretion.

It should also be noted that other in vivo animal models can provide strong modeling of CODE mutations. As a higher animal model, genetically modified MYO5B(P663L) pigs demonstrated a strong MVID phenotype similar to P660L mutants in human Navajo MVID patients (Engevik et al., 2020). As in Navajo MVID patients, the mutant newborn pigs demonstrated profound diarrhea, microvillus inclusions, reduced brush border transporters, and the maintenance of apical CFTR (Engevik et al., 2020). Lower vertebrates such as zebrafish have also proved useful for modeling disease. In particular, zebrafish mutations in the MYO5B homolog *goosepimples* (*gsp*) in zebrafish show microvillus atrophy, microvillus inclusions, and subapical accumulation of secretory vesicles as is seen in MVID patients and mammalian models (Sidhaye et al., 2016).

While the use of knockout mice to model loss-of-function mutations has been extremely productive, many patients with monogenic diseases possess a variety of homozygous missense mutations or compound heterozygous point mutations. These include either two different missense mutations or a combination of one missense allele with an early termination allele. In MVID and other CODE disorders, it remains unclear that loss-of-function missense mutations always produce the same pathophysiology. Indeed, while the effects of mutations on protein function are often predicted by molecular modeling software, only one MVID-causing MYO5B mutation has ever been shown as disabling experimentally, the rigor mutation status of the Navajo P660L mutation (Knowles et al., 2014). Thus, the molecular impacts of most mutations have not been tested directly. Gene-editing systems including CRISPR/Cas9 now allow directed sequence modifications in experimental animals. One problem with performing these gene-editing approaches is that both mice and pigs with homozygous mutations often show prenatal or early postnatal mortality. To circumvent this issue, recent studies have utilized a modified CRISPR/Cas9 approach to purposefully develop mice with heterozygous alleles through the injection of CRISPR/Cas9 reagents into only one cell of two-cell stage embryos (Burman et al., 2023; Wu et al., 2019). These chimeric mice can then be bred with wild-type mice to give rise to heterozygote progeny, which can then be crossbred with inducible knockout mice to allow the evaluation of the patient-derived mutation allele in adult mice. This approach was used to model a patient-specific compound heterozygous mutation in MYO5B, where one allele had a missense mutation (p.G519R) and the other allele carried an early truncation mutation (*Vil1-Cre*^*ERT2*^;*Myo5b*^*flox/G519R*^). These mice were shown to authentically recapitulate the patient’s phenotype after tamoxifen injection (Burman et al., 2023). Interestingly, compared with biallelic MYO5B deletion or the P660L mutation, this MVID model showed MYO5B(G519R) colocalized with RAB11A in abnormal subapical vesicles in enterocytes, a pattern that was also observed in the patient tissues (Burman et al., 2023). Thus, specific modeling of patient mutations in mice can provide variant-specific information that could affect disease severity as well have utility in assessment of patient-specific responses to putative treatments.

### Modeling disease in vitro with patient-derived enteroids and organoids

The use of patient-derived enteroids generated from intestinal epithelial stem cells as models for disease has expanded rapidly since their initial introduction and characterization (Sato et al., 2009). Enteroids generated from intestinal crypt stem cells, sometimes also referred to as “intestinal organoids,” can contain all differentiated and proliferative epithelial populations and display similar cycling kinetics to that seen in vivo. Enteroid cultures without mesenchymal cells or luminal microbiota exhibit autonomous epithelial cell physiology and pathophysiology, which more closely resembles the native tissue due to similar dynamics of self-renewal and mature enterocyte differentiation in culture. This along with appropriate localization and levels of enzymes and transporters at the epithelial apical brush border makes enteroids a valuable model system for studying both normal and CODE disorder cell biology (Foulke-Abel et al., 2016). Enteroids from normal mice and mutant mouse strains can be used to model specific disease in vitro. Mouse enteroids have been utilized extensively to study the aspects of cell physiology in association with MYO5B loss (Engevik et al., 2018; Kaji et al., 2020).

Recently, patient-derived enteroids have been utilized to gain insights into intestinal disorders. Obtaining these patient-derived enteroids requires a considerable coordination between gastroenterologists and research laboratories since endoscopic procedures may only be performed at the time of diagnosis in many of these infants, who are often extremely ill. In addition, the initial establishment of disease enteroids is often more difficult than for enteroids derived from normal tissues. In the case of MVID, these enteroids derived from patient duodenal biopsies can be used to evaluate various aspects of disease with specific genetic mutations. Human enteroids can be studied in three-dimensional Matrigel cultures as spheroids with differentiation stimulated by the removal of Wnt activators from the media. Furthermore, these enteroids can be grown as monolayers with stimulation of maturation and differentiation through the withdrawal of Wnt and a conversion to air-liquid interface (ALI) culture (Foulke-Abel et al., 2016). As shown in Fig. 2, ALI enteroid cultures develop a dense apical brush border, similar to normal enterocytes in vivo. Just as importantly, the mature enterocyte monolayers can be studied for proper apical polarization and delivery of transporters by electrophysiological assessment utilizing classical Üssing chambers and intracellular pH measurements as well as immunofluorescence staining for transporters and brush border proteins (Hasan et al., 2021; Kalashyan et al., 2023; Yin et al., 2018).

As noted above, disabling mutations in some CODE disorders such as DGAT1 cannot be modeled in mice. This has made modeling of DGAT1 mutations in patient-derived enteroids a priority for this disorder. Indeed, the enteroids generated from DGAT1 deficiency patients also recapitulate the alterations in brush border and in lipid trafficking observed in the children (van Rijn et al., 2018). These human patient-derived enteroids provide critical systems for detailed evaluation of the cell biology of human diseases.

## Studying congenital diseases leads to insights into cell biology

**Figure 3.**
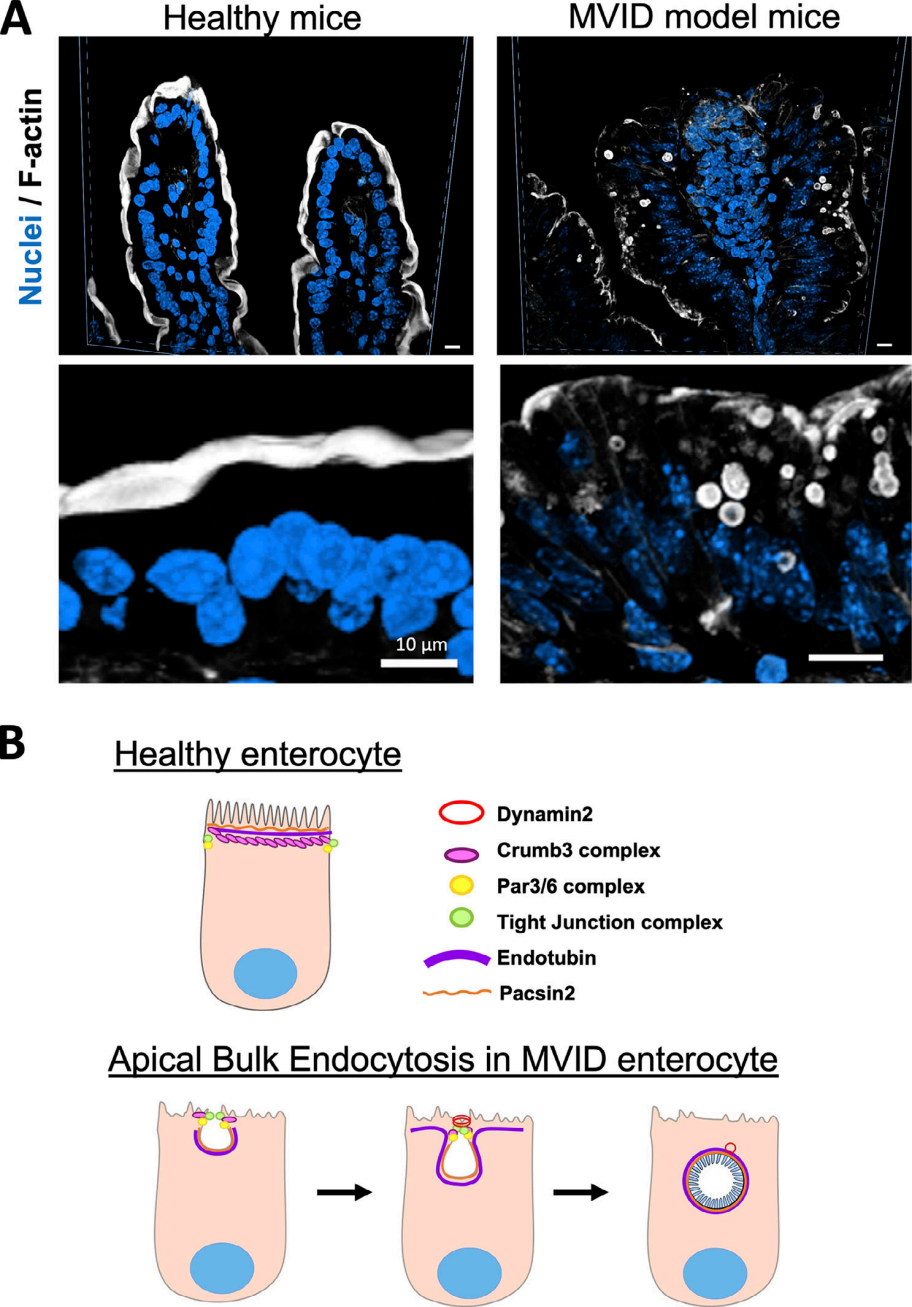
**Identification of apical bulk endocytosis in MYO5B knockout enterocytes. (A)** MVID model mice (MYO5B knockout) show loss of actin-rich apical microvilli and formation of microvillus inclusions. Examination of these MYO5B KO mice showed that microvillus inclusions were formed through a process of apical bulk endocytosis. **(B)** Schematic summarizes the process of apical bulk endocytosis, which requires Pacsin 2 and dynamin 2 and involves the recruitment of tight junction proteins to the site of apical bulk endosome scission.

A conundrum in MVID pathophysiology over the years has been the origin and relevance of microvillus inclusions themselves. In patients, the number of inclusions varies widely from none to many. In mouse models, more microvillus inclusions are observed in the proximal intestine compared with the distal (Weis et al., 2016). Given the large size (often >5 µm) of inclusions, many considered that the presence of large round vesicles with mature microvilli indicated that these inclusions represented failed apical fusion events. Nevertheless, both the mouse models and derived enteroids have allowed us to demonstrate that the microvillus inclusions formed, primarily in the lower villus enterocytes, through a previously unrecognized process of apical bulk endocytosis (Engevik et al., 2019) (Fig. 3). This process involves invagination of the apical membrane and subsequent dynamin-mediated scission. Importantly, in mice with dual knockout of MYO5B and PACSIN2 (syndapin 2), a regulator of membrane curvature (Dharmalingam et al., 2009), even though no inclusions formed, the mice still showed deadly diarrhea and loss of apical sodium transporters (Engevik et al., 2019). These results suggest that the formation of microvillus inclusions by apical bulk endocytosis is not a critical part of the pathology of MVID, but rather revealed a previously unrecognized trafficking pathway. Further studies have demonstrated that the process of inclusion formation requires the recruitment of tight junction proteins that appear to seal the region of endocytosis (Engevik et al., 2021). Basolateral junction structures remain intact in all studied MYO5B knockout mouse intestines, distinguishing MVID from leaky gut syndromes. Although it remains unclear whether the loss of MYO5B exaggerates the apical bulk endocytosis process, microvillus inclusion formation is usually more prominent in neonatal mice compared to adult-induced models. Previous studies have suggested that neonatal enterocytes may use apical endocytosis of luminal contents as a mode for rapid nutrient uptake necessary in the developing and maturing intestinal mucosa postnatally (Cox et al., 2017; Wu et al., 2021). Thus, apical bulk endocytosis may be a physiological pathway for apical uptake that is amplified upon MYO5B loss.

A few discrepancies in apical protein trafficking pathways have been noted among different MVID models that indicate the power of comparative cell biology. In one example, the apical localization of overexpressed tagged CFTR in CaCo-2 cells was affected by MYO5B loss (Vogel et al., 2015). In contrast, duodenal tissues of multiple MVID patients and MYO5B deficient mice consistently demonstrate that CFTR expression is maintained on the apical membrane (Engevik et al., 2018; Kravtsov et al., 2016). These results suggest that over-expression of transporters in cancer-derived cultured cells may not always accurately recapitulate in vivo function.

## Translating cell biology into treatment

**Figure 4.**
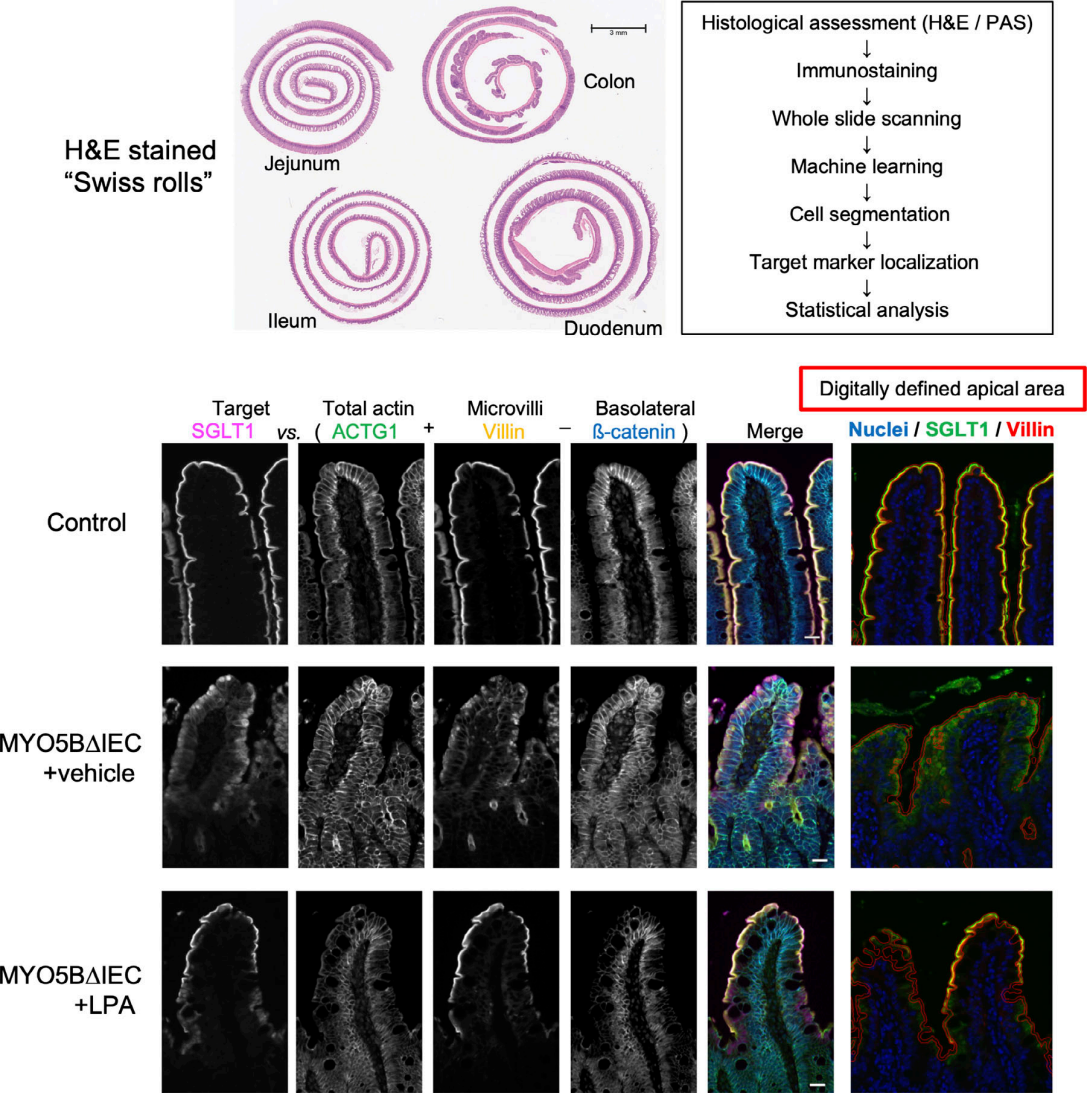
**Evaluating the effects putative treatment in mouse models of MVID.** Since disease in MVID affects the entire intestine, examination of the entire length of small intestine is required. The development of digital analysis tools to examine the localization of transporters (in this case SGLT1) in the villin and gamma-actin (ACTG)-rich brush border membranes allows quantitative evaluation of the effects of LPA treatment on the inducible, intestinal-targeted MYO5B knockout mice (MYO5BΔIEC). The panels at right demonstrate overlay images of SGLT1, villin, and apical area (red line) defined by the image math before and after treatment with lysophosphatidic acid (LPA) as a measure of treatment success. Scale bar = 20 µm.

The availability of mouse models for MYO5B loss-of-function and their derived enteroids along with patient-derived enteroids has allowed examination of possible therapeutic strategies to ameliorate the diarrhea in MVID (Fig. 2). Initial studies evaluated the ability of lysophosphatidic acid (LPA) on apical trafficking in MYO5B-deleted mouse intestines and in mouse enteroids (Kaji et al., 2020). LPA is an endogenous signaling lipid that can activate G-protein-coupled receptors on a number of epithelial cell types (Lin et al., 2010). LPA treatment improved the delivery of NHE3 and SGLT1 to the apical membranes of MYO5B KO mouse enterocytes, reestablishing functional sodium transport (Kaji et al., 2020) (Fig. 4). These studies indicate that small molecule activation of LPA receptors could bypass the blockade to trafficking associated with MYO5B loss. Further studies demonstrated that MYO5B knockout mice show a marked loss of Wnt signaling molecules concomitant with continued Notch signaling (Kaji et al., 2021). These studies suggested that the loss of MYO5B may result in an imbalance in Wnt/Notch signaling leading to a deficit in proper maturation of enterocyte lineages as they traverse the crypt regions and exit into the villi. This mechanism may also underlie other congenital diarrheal diseases, since e EPCAM knockout mice, which model the congenital tufting enteropathy (CTE) (Sivagnanam et al., 2008), also show increased Notch signaling that interrupts stem cell differentiation (Das et al., 2021). To test whether rebalancing of the Wnt/Notch pathways could alter the pathophysiology of MYO5B loss, a Notch signaling (gamma-secretase) inhibitor was administered to intestinal-targeted MYO5B knockout mice. A gamma-secretase inhibition elicited a marked improvement in ion transporter localization on the apical membranes of enterocytes (Kaji et al., 2021). These investigations established that the promotion of differentiation of nascent enterocytes can ameliorate the effects of MYO5B loss.

To our knowledge, only one report has utilized human patient-derived enteroids from MVID patients to characterize brush border function and evaluate putative treatments to reestablish a functional brush border (Kalashyan et al., 2023). Consistent with mouse model studies, gamma-secretase inhibition improved brush border maturation and NHE3 function patient-derived enteroids from two different patients with either total loss of MYO5B or the previously described P660L variant (Kalashyan et al., 2023). Furthermore, the inhibition of calcium-activated chloride channels (CaCCs) in addition to CFTR in patient-derived enteroids demonstrated a significant anti-secretory effect (Kalashyan et al., 2023) reflective of a more significant and age-dependent contribution of this pathway in pediatric enteroids. In addition, given that CaCC function is not the predominant chloride pathway involved in fluid secretion in mouse small intestine, these studies provide a rationale for the use of age-specific human enteroids as tools to screen anti-diarrheal drugs for CODE disorders. One can expect that similar strategies to test potential therapeutics using patient-derived enteroids from DGAT1 deficiency patients (van Rijn et al., 2018) will accelerate preclinical efforts. In general, these recent studies have highlighted the potential of patient-derived organoids to facilitate functional and histological evaluation of transporter physiology and enable efforts to develop more effective treatment strategies.

### Personalized cell biology and the development of effective treatments for monogenic diseases

The preceding discussion using MYO5B-mediated MVID as a case study has provided a glimpse at how the development of model in vitro organoid and in vivo animal models can facilitate a greater of understanding of monogenetic GI disease pathophysiology and illuminate the key aspects of epithelial cell trafficking. Despite these studies, several major gaps remain for practically moving cell biological insights into the treatment of patients. First, almost one hundred different mutations in MYO5B have been identified in human patients, but only one (P660L) has been molecularly characterized. Thus, while many of the mutations have implied impacts on motor function, their true influence has not been fully characterized. Although some MVID patients eventually develop liver disease, several MYO5B mutations within or around the motor domain (e.g., C266R) have been identified as variants that produce liver-specific disease without significant intestinal manifestations (Gonzales et al., 2017; Qiu et al., 2017). This suggests that there is a clear gap in knowledge of the cell- and organ-specific functions of MYO5B, since mutations affecting the motor domain function would be expected to produce similar effects across epithelial cells. Thus, a detailed examination of the effects of particular mutations within the correct cellular context remains a priority for our understanding of the phenotypic heterogeneity related to alterations in MYO5B function.

Second, while human organoids represent a powerful model system for understanding genes important for epithelial function and for putative treatments, they provide information on only epithelial autonomous mechanisms. It is unclear that in some disorders whether interactions with stromal and immune mechanisms or even with the luminal microbiome may contribute significantly to the final disease phenotype. This type of integrative biology may be addressed in more detail in in vivo animal models.

Third, in the context of monogenetic mutations that are manifest only in individuals with either homozygous or compound heterozygous mutations, reintroduction of the expression of wild-type protein should reestablish the wild-type phenotype. This scenario would then be amenable to technologies such as nanoparticle delivery of mRNA coding for the wild-type protein. Clearly, the use of RNA nanoparticles has received expanded interest following the development of effective vaccine strategies against COVID-19 (Ye et al., 2023). Thus, while the use of small molecule therapies has broad deliverability, the reestablishment of wild-type proteins will require targeting to the affected enterocytes or even to progenitor cell populations. Given the large target organ area in the intestines, efficient and effective delivery of nanoparticles may prove difficult. However, since CODE disorders affect surface intestinal epithelial cells, there may be comparative advantages for disease-targeting by oral delivery systems that do not require significant systemic uptake. Current delivery methods may be more promising in the context of liver disease, where organ uptake may be greater, and the repair of smaller regions could ameliorate cholestasis. Still, human enteroids provide a promising method for initial testing of mRNA therapy efficacy, and the availability of mouse models similarly provides an in vivo preclinical testing system that can facilitate development of appropriate delivery systems.

In summary, the investigation of monogenetic diseases such as CODEs offers a unique opportunity to uncover insights into basic cell biology and physiology. Modeling of disease in both patient-derived organoids and animal models has allowed application and translation of some of these basic cell biological insights into potential treatment strategies and provides hope for improving outcomes in children with these debilitating genetic disorders.
